# Analyzing some concepts of immune regulation of the last three decades: Fostering greater research resilience despite the information overload. A personal view

**DOI:** 10.3389/fimmu.2022.960742

**Published:** 2022-11-02

**Authors:** Peter A. Bretscher

**Affiliations:** University of Saskatchewan, Saskatoon, SK, Canada

**Keywords:** immune regulation, self-nonself discrimination, immune class regulation scientific progress, science policy, philosophy of science, information overload

## Abstract

There is considerable interest in whether increased investment in science, made by society, pays dividends. Some plausibly argue the increased rate of production of information results in an ossification of the canon. Reports, challenging the canon, fall by the wayside. The field thus becomes increasingly complex, reflecting not so much the reality of nature but how we investigate the subject. I suggest that focusing on and resolving the paradoxes evident within a canon will free the logjam, resulting in more resilient research. Immunology is among the fastest growing of biological sciences and is, I suggest, an appropriate case study. I examine the commonly accepted frameworks employed over the last three decades to address two major, related immunological questions: what determines whether antigen activates or inactivates CD4 T cells, and so whether immune responses are initiated or this potential ablated; secondly, what determines the Th subset to which the activated Th cells belong, thus determining the class of immunity generated. I show there are major paradoxes within these frameworks, neglected for decades. I propose how research focused on resolving paradoxes can be better fostered, and so support the evolution of the canon. This perspective is pertinent in facing critical issues on how immune responses are regulated, and to more general issues of both the philosophy of science and of science policy.The last section is in response to questions and comments of the reviewers. It brings together several considerations to express my view: the same frameworks, formulated in response to the two questions, are useful in understanding the regulation of the immune response against model antigens, against self and foreign antigens, those of tumors and of pathogens.

## Introduction

Many commit to science in the belief it is a relatively objective means of gaining insight. Society invests heavily in science on this belief. I have been an immunologist for over fifty years. I feel the culture supporting immunological research has changed dramatically over this period in a manner that makes me uneasy. This change reflects the problem posed by the information overload (1). The number of papers published in most scientific disciplines has increased about 16-fold over the last fifty years (2). Neither are contemporary papers as accessible as those of fifty years ago. My thoughts primed me to respond to Chu and Evans’ recent paper, entitled “Slowed canonical progress in large fields of science” (3).

## Research investment and productivity

Chu and Evans argue, from an analysis based on papers published in diverse scientific fields, that, as research investment increases to high levels, the increased rate of information produced is stultifying. Not only does progress not increase proportionally but the nature of the progress changes. It becomes more difficult to challenge the canon. They employed parameters, in analysing 90 million scientific reports, to assess research resilience. One parameter employed measures how new papers penetrate the field, as measured by their relative citation, as research intensity increases. Penetration decreases overall as research intensity increases. The field becomes ossified. The implication is that potentially *significant* challenges to the canon are often not recognized (3).

It may be difficult to develop prescribed criteria for judging “significance”. I address below thoughts on how some potentially “significant” research proposals can be identified and their funding fostered, to thereby increase research resilience.

Chu and Evans developed a vivid analogy to illustrate what they believe happens. When single grains of sand are occasionally dropped onto a sandpile, the grain normally lodges where it lands; only occasionally does an additional grain of sand cause an avalanche. When grains are dropped more frequently, such that one grain lands before the consequences of its immediate predecessors have been fully realized, smaller avalanches continuously occur. A grain represents a publication, the sandpile the state of the field, the rate of bombardment the rate of information delivery, and the size of the avalanche the impact of the paper on the field (3).

What resilience refers to in Chu and Evans’ analysis is ambiguous, as the parameters reflecting resilience are not assessed for different types of papers. The authors note the most cited paper over years in “molecular biology” reports a methodology. It would be significant to explore whether penetrance is different for different types of paper, for example those reporting new methodology and those challenging the canon. The authors suggest that low penetrance of new papers includes those challenging the canon (3). I agree with the plausibility of this. This assessment is supported by the accumulation of neglected paradoxes (1).

## Immunology as a field of intense research

The change in research culture over the last fifty years can be partly explained by Chu and Evans’ analysis. Despite truly remarkable advances, the immune system is regarded by most researchers as ever more complex. Textbooks are getting much larger, not smaller. The authors suggest the canon would, with greater time for reflection by practicing scientists, have evolved to achieve greater explanatory power. The field would therefore appear less complex. How might “inappropriate” complexity be diagnosed? Niels Bohr, the theoretical physicist, said: “How wonderful that we have met with a paradox. Now we have some hope of making progress” (4). I suggest that a sign of the ossification Chu and Evans describe is when frameworks commonly employed appear inconsistent with observations, and/or appealing principles, and such paradoxes are ignored by the research community. Paradoxes focus attention on foundational issues. In my opinion, certain areas of contemporary immunology provide a suitable case study for what happens as research intensifies. I suggest, based on such an examination, how research resilience may be restored (1).

## Immunology as a case study

Two interrelated, basic questions address how immunity is regulated. Valid answers to these have implications for medical interventions. I outline these questions and the commonly accepted frameworks, employed in the last thirty years, to address them. My purpose is to distil why these frameworks are unsatisfactory, as reflected by neglected paradoxes. This analysis provides a scenario for considering how ossification of the field may be ameliorated. I naturally choose questions I am familiar with, as I have attempted to address them myself (5, 6). However, my focus here is to point out the prevalence of neglected paradoxes and their potential use in fostering progress, not to justify in detail my personal views.

### Question 1: How does antigen inactivate and activate CD4 T cells?

Most immature lymphocytes, with the capacity to respond to self-antigens, are eliminated by self-antigens in the primary lymphoid organs, as generated (7). This elimination results in “central tolerance”. A minority of self-antigens, such as insulin, are insufficiently present in primary lymphoid organs to cause “complete” central tolerance (8). There is a need for “peripheral tolerance”. Antigen can interact with peripheral, naive lymphocytes to either inactivate or activate them (9). It is natural to expect that “peripheral antigens”, such as insulin, normally inactivate their lymphocytes, whereas foreign antigens activate theirs.

Cohn and I proposed in 1970 the one lymphocyte/multiple lymphocyte model for the antigen-dependent inactivation/activation of lymphocytes (10). Peripheral antigens are present before mature lymphocytes begin to be generated (7, 11). They therefore inactivate their mature lymphocytes as generated, one at a time. Lymphocytes specific for a foreign antigen, F, accumulate in its absence; when F impinges upon the immune system, it can mediate lymphocyte interactions to generate immunity (10).

Many subsequent observations fit this model (12, 13). The activation of most B cells to produce antibody, and of most CD8 T cells to generate cytotoxic T lymphocytes (CTL), requires activated CD4 T helper cells; in the absence of such help, antigen inactivates the B and CD8 T cells (12, 13). The first basic question is: what determines whether antigen inactivates or activates CD4 T cells?

Our 1970 model (10) and its modern version (14), and the models respectively initiated by Janeway (15) and Matzinger (16) in 1989 and 1994, all state, as now understood, that a CD4 T cell, only receiving an antigen-dependent signal, is inactivated. All also state that the activation of the CD4 T cell requires in addition a second, critical signal. According to Janeway’s model, this signal is initiated when a pathogen-associated molecular pattern (PAMP), present on the antigen or associated with its delivery, is recognized by pattern-recognition receptors (17). According to Matzinger’s model, this signal is generated under dangerous circumstances, as assessed by the recognition of a danger-associated molecular pattern (DAMP) (18). According to our 1970 model (10, 14), this signal is generated following the recognition of antigen by another lymphocyte, a T helper cell. Our model provides as explanation for peripheral self-nonself discrimination, as outlined above. Janeway’s model means the immune system really discriminates non-infectious from infectious entities, and Matzinger’s model antigens impacting under non-dangerous from dangerous circumstances. I indicate below paradoxes that make me question the PAMP/DAMP models.

### Question 2: What controls whether antigen induces cell-mediated immunity or IgG antibody?

The immune system has different means, reflecting different classes of immunity, to fight foreign invaders. It also has a decision-making process as to which to deploy. Cell-mediated immunity and IgG antibody are the two main classes. We simplify by considering only these two. This is sufficient to describe the principal frameworks, and issues arising from them.

A naive CD4 T cell can be activated to give rise to Th cells belonging to different Th subsets. Exclusive cell-mediated immunity is correlated with Th1 cells, and of IgG antibody production with Th2 cells (19). Thus, the second question can be recast: what determines whether antigen induces a peripheral, naïve CD4 T cell to give rise to Th1 or Th2 cells?

### The decision criterion controlling the Th1/Th2 phenotype of a response

The most prevalent ideas are that PAMPs/DAMPs are not only required to activate CD4 T cells, but their nature, associated with the antigen inducing the response, and circumstances of immunization, determine the Th1/Th2 phenotype of the response (20–23).

My 1974 Threshold Hypothesis is an alternative proposal (24, 25). It assumes the activation of a CD4 T cell requires antigen mediated CD4 T cell interactions; a threshold of signals arising from weak interactions results in Th1 cells, and a threshold of signals arising from robust interactions in Th2 cells. This threshold mechanism accounted for the variables of immunization known in 1974 to affect the Th1/Th2 phenotype of the ensuing response. Minimally foreign antigens, for which there are relatively few CD4 T cells, only generate Th1 cells (26); low, suboptimal doses of more foreign antigens only induce Th1 cells (24, 27–29); a moderate dose first generates Th1 cells and, as helper T cells multiply, the response often evolves towards a Th2 mode (24, 27, 29); an even higher dose, optimal for supporting CD4 T cell interactions, rapidly evolves into a Th2 mode (24, 27, 29). These patterns are universally observed, when appropriately examined, including infection by HIV (30).

The hypothesis also makes a unique prediction: partial depletion of CD4 T cells, at the time of immunization, modulates the response from a Th2 towards a Th1 phenotype (25).

## Paradoxes

I do not favour the PAMP/DAMP proposals described above, in part because there are several paradoxes within their context. I outline five below. These either reflect striking and paradoxical observations made in one system (#2) or they represent striking and paradoxical experimental generalisations.

1. Many foreign, vertebrate, non-PAMP bearing antigens, administered under non-dangerous circumstances, are highly immunogenic, rather than inducing unresponsiveness. Examples are foreign, vertebrate red blood cells delivered with a very sharp needle (29, 31).
2. Mouse cytochrome C (MCC)-specific CD4 T cells can be raised in mice. Immunization with MCC in complete Freund’s adjuvant (CFA) is ineffective, even though CFA contains dead, PAMP-expressing mycobacteria. Janeway noted the utility of CFA in raising immunity to otherwise non-immunogenic antigens in formulating his model (15). This observation is paradoxical within Janeway’s framework. Moreover, MCC-specific Th cells are only induced if, in addition to immunization with MCC in CFA, they are given MCC-specific activated B cells. These observations support my proposal that activation of CD4 T cells requires CD4 T cell cooperation mediated by an antigen-specific B cell (14, 32).
3. The dependence of the Th1/Th2 phenotype of immune responses on antigen dose is not readily explained by the PAMP/DAMP proposals. Moreover, this dependence holds for foreign, vertebrate, PAMP-free antigens (29, 31), and for responses to PAMP-expressing protozoa (33, 34) and PAMP-expressing mycobacteria (35, 36). A PAMP-independent explanation for this dependence seems called for.
4. The evolution of the response from an exclusive Th1 towards a Th2 mode, after antigen impact, is true of responses to foreign, vertebrate, PAMP-free (28, 29) and PAMP-containing antigens (34, 36). This evolution is paradoxical even when only considering PAMP-expressing antigens, as the PAMPs do not change with time after antigen impact.
5. Partial depletion of CD4 T cells, around the time of immunization, modulates the response from a Th2 towards a Th1 phenotype, all other variables being kept the same (37–41). This is paradoxical as such depletion is not expected to change the PAMP/DAMP signals.

## Medical interventions

The immune system is involved in allergies, autoimmunity, cancer, infectious diseases, and transplantation. The two questions discussed are pertinent to interventions in these medical fields (5, 6). We illustrate their importance by outlining the potential significance of recognizing that antigen dose is critical in determining the Th1/Th2 phenotype of the response. This generalization is incomprehensible in the context of the PAMP/DAMP frameworks, as just outlined. Thus, the findings described below are paradoxical in the context of these frameworks.

Some pathogens are only/best contained by a Th1 response; chronic/progressive disease is often associated with a substantial/predominant Th2 component of the response. Current vaccination protocols enhance Th2, IgG antibody responses, and so are ineffective against these pathogens (6).

Repeated immunization with low doses of an antigen leads not only to Th1 responses but can lock the response into a Th1 mode, as Parish showed in the late 1960s (28). Our low dose vaccination strategy is based on Parish’s findings. Infection/inoculation with low numbers of protozoan parasites (33, 42), of mycobacteria (35, 36) and of cancer cells (43) generates a stable Th1 response and Th1 imprint. Challenge of such exposed mice to a larger infection/inoculation, that results in a Th2, IgG antibody response and “disease” in naïve mice, results in a stable Th1 response and resistance. We and others have also shown, in patients with visceral leishmaniasis, that reducing the antigen load, by administering anti-parasitic drugs, modulates the on-going immune response from a Th1/Th2 to a Th1 phenotype, resulting in resistance to reinfection (44). It appears that appropriate lowering of antigen levels can thus be employed for treatment.

These studies seem pertinent to vaccinating against HIV-1 (45), and for immunotherapy of HIV-1 infected individuals (46) and of tuberculosis patients (47). Buddle showed cattle can be made resistant to experimental tuberculosis by reducing the “standard dose” of the vaccine by a million-fold (48). I am surprised that his and our studies, given their potential medical significance, have not better penetrated the literature over the last two/three decades. One likely reason is that the low dose strategy has no rationale in the context of the widely held PAMP/DAMP hypotheses.

## Conclusion: Fostering research resilience

I have participated on research panels of different kinds. I have chatted science with immunologists and researchers in related fields. These experiences have led me to appreciate certain tendencies.

A non-specialist is more open to knowledge he/she is unaware of. Most contemporary immunologists, as I have assessed through discussions, do not know of Parish’s work of the late 1960s on Th1 imprinting, the basis of our low dose vaccination strategy. I have the impression that most immunologists assume, if this was a valid generalisation, they would surely know of it. I have listed above some paradoxes in the context of widely accepted frameworks. Most specialists appear to find it harder to share Bohr’s enthusiasm for paradoxes than do non-specialists. This is not only because of vested interests. A specialist is aware of the cost of abandoning concepts that have appeared useful in the past. A non-specialist has something like the freedom of a child in considering matters ab-initio.

I have come to appreciate that the nature of the discussion by research panels depends upon whether different panel members have different specializations and the degree to which a genuine consensus is sought. I propose, based on my experiences, how research resilience in research-intensive fields can be fostered, and how the efficacy of my proposal can be assessed.

Most grants for medical research in Canada are awarded by the Canadian Institutes for Health Research. My thoughts are cast within the context of how these institutes make awards. Members of a grant panel all have expertise in a particular field. Those members, chosen to judge a proposal, often have more focussed expertise, “appropriate” to the proposal. This is the “conventional panel”. I suggest there should be a second, alternative panel. Panel members would also be successful researchers, but most in fields neighboring the field of the proposal. Every proposal would be assessed by some non-specialists and usually by a specialist, sometimes leading, I suspect, to interesting and real discussions. Applicants could choose to which panel they apply. Some would appropriately choose the conventional panel. Others could choose the alternative panel, particularly if their proposal challenged the current canon. It is possible to explain to the non-specialist basic ideas in even as complex a field as immunology.

Excellent proposals would be received by both panels. Those with a potential of leading to valuable, disruptive research are more likely to be submitted to and funded by the alternative panel. Whether the alternative panel funds more impactive research could be assessed. The parameters employed by Chu and Evans to measure research resilience could be applied over time to papers supported by grants from the two panels and compared (1).

I wish, in closing, to briefly comment on Chu’s response (49) to my proposals, as expressed in my very brief letter (1) to PNAS, in which Chu’s paper was originally published. Chu acknowledged my proposals may increase funding of research outside the canon. However, he ignored my suggestion that paradoxes can focus on research that challenges and helps the canon evolve. He also pointed out that ossification of the canon affects not only research funding, but publishing, academic promotion, and invitations to meetings. He expressed reservations about the efficacy of my proposals. I suggest the installation of a two-tier reviewing system for publications, similar to the two-tier reviewing system of research proposals, may help ameliorate the logjam. Such innovations are likely to result in time in changes in assessing promotion and the reputations of individuals.

## In response to reviewers’ comments

I am grateful to both reviewers for their substantial and considered comments. Neither reviewer challenged the validity of the paradoxes I described. Both brought up observations that they thought might be difficult to account for in terms of the frameworks I favour. I think these questions are particularly valuable as they likely reflect the kinds of questions the general reader would raise. In addition, they are naturally also questions I had asked myself, given the frameworks I favour. I decided to respond to the reviewers’ comments in a separate section, rather than by modifying the original text. Such a modification would have resulted in a disjointed text, and the loss of focus on the importance of paradoxes, the aim of the original submission. Both reviewers also brought up philosophical points. I respond to these before discussing the significance of the observations they raised.

One reviewer suggested my frameworks may fail the criterion of being falsifiable, a criterion that the philosopher Karl Popper suggested should be satisfied by any worthy scientific idea (50). I understand Popper came to this criterion when he encountered proponents of the three competing psychologies of Freud, Jung, and Adler. According to Popper, all proponents claimed they could account for any psychological observation. This ability was not a virtue, according to Popper. He suggested a “significant theory” should be sufficiently “concrete” or “robust” that it makes clear predictions that can be tested and may be shown to be wrong; in other words, a theory should be falsifiable. I think this aspect of Popper’s philosophy of science has merit. However, from this perspective, I believe the two frameworks I have put forward make predictions that have been successfully tested and could have been falsified. I therefore do not think they suffer from the criterion of not being falsifiable. In addition, they are sufficiently robust to be useful in proposing and testing diverse strategies of medical intervention (5, 6). I have often felt the Danger Model of CD4 T cell activation to be a very flexible idea, as danger is so loosely defined. However, the fact that paradoxes can be envisaged within its context, as outlined above, shows it too can be falsified.

I was asked what are frameworks and how are they generated? The view was expressed that frameworks appear to be highly subjective structures. I think the latter opinion calls for a response.

A framework refers to any proposed conceptual scheme, or working hypothesis, independently of its plausibility. Its plausibility changes with time and is different in the minds of different researchers at a given time.

Frameworks are generated in response to one or more questions. Their plausibility depends on whether they can give a logically consistent answer to the question(s) consonant with the pertinent and available evidence.

A consideration of whether frameworks are subjective calls for a few preliminary remarks. Observations and concepts exist at different levels, at the level of the system, of cells and of molecules. Frameworks at a “higher” level (system>cellular>molecular) are very often used to interpret the significance of observations/considerations made at a lower level. The plausibility of an interpretation at a lower level usually depends on this relationship, independently of whether the interpreter is conscious or not of this relationship. An obvious and outstanding example is the molecular structure of DNA, that contains an explanation of how the hereditary material can replicate, an attribute at the level of the system. An appeal of the DAMP/PAMP frameworks are their provision of a context for interpreting cellular and molecular observations in a way that addresses how “peripheral tolerance” and immune class regulation are achieved. I of course argue their implausibility on the basis of paradoxes. An example of what I consider a misleading framework is provided by the Cytokine Implementation Hypothesis, a system-level idea. An individual entertaining this framework, and knowing IL-4 is necessary for the generation of Th2 cells (51), may seek the non-T cell source of the IL4. Several well-known publications have been inspired by such considerations. For example, it has been found that mast cells are required in certain circumstances to generate Th2 cells (52). I would not dispute such particular findings. However, the significance often drawn from such observations is that IL4, from a non-T cell source, is in general required to generate Th2 cells. If believed to be true, and this generality is incorrect, it can be an impediment to progress. In particular, this view leads to a focus on seeking the source of the IL4, rather than other critical events determining whether Th2 cells, over Th cells of other subsets, are predominantly generated. I will return to this subject in response to further comments from the reviewers.

All frameworks, being constructed by people, are subjective. I do not find the subjective/objective distinction that useful in assessing frameworks. I find the concept of plausibility more apt.

The simpler a conceptual scheme, the more “robust” it is in making predictions, and the broader its scope in “accounting for” observations and addressing conceptual questions, without incurring paradoxes, the more *plausible* it is. A requirement for certainty, or objectivity, if sincerely sought, is destructive, as unattainable. To achieve a plausible framework, one must seek and transcend paradoxes. This is why Bohr and I think paradoxes so valuable. Our frameworks are admittedly always provisional and never complete. The Clonal Selection Theory is highly plausible but does not provide a complete description of how the immune system functions, or of the world.

In the 1960s and 1970s most experiments were “hypothesis driven”, whereas today many studies are “data driven”. A totally new field of bioinformatics has emerged, that many regard as data driven. One of the reviewers requested my perception of how these changes could be reconciled with the ideas I was developing on the importance of paradoxes.

The faith in the value of data is a hallmark of our times. The underlying thoughts of some that objective and significant statements can be made without a context has inspired much misguided philosophy of science, in my opinion, as exemplified by The Vienna School of Logical Positivists of the early 1900s and their followers. Referring to an observation as an observation reflects an awareness that “the observation” was made by a process whose legitimacy, in terms of the way it was made or of its interpretation, may be open to doubt. The use of the word data carries with it a much more insistent tone. Observations may be “incorrect” but not data! This belief is particularly prevalent and strong when the “data” are of a molecular nature, as there is such a substantial understanding of how atoms and molecules function. So how could such data be misleading? We all most want to understand how the immune system functions as an integrated organ. So how do we go from observations/considerations at the molecular/cellular level to those at the higher level of the system? A couple of examples are enlightening.

Burnet and Fenner proposed in 1949 that the early presence of self-antigens in ontogeny, i.e. in the early history of the animal, results in tolerance towards them (53). Billingham et. al. tested this idea; exposure to certain foreign antigens early in development resulted in the exposed animals, as adults, being unable to immunologically respond to these antigens in the manner that unexposed animals could (54). Although Billingham et. al. employed certain foreign antigens, virtually all accepted their observations as evidence supporting Burnet and Fenner’s conjecture, a very general statement concerning tolerance to all self-antigens. This example illustrates that the general significance attributed to the particular observations of Billingham et al depends upon ideas at the level of the system. This is a good example of the “old style” of immunology of “working hypotheses”.

Recent conversations with immunological colleagues have made me realize that many today think the purpose of science is to record objective facts. However, “facts” are only important if there are grounds to believe they have general significance and so can be interpreted in terms of generalizations or concepts at the level of the system. Many regard molecular facts as indisputable and so the holy grail of science. I now provide an example of how molecular observations have been misleading and indicate why they are misleading when not examined in the context of observations and/or considerations at a higher level.

The example I have chosen is particularly interesting because so biologically consequential. In the context of our 1970 two signal model of lymphocyte activation (10), it became widely believed that signal 1, that leads to B cell inactivation when generated alone, is initiated when surface Ig (sIg) is crosslinked by antigen. The observations of the mid-1970 that led to this conclusion was that antibody, cross-linking sIg of B cells, stimulated them to divide (55). This model is still widely believed. I have been most uneasy about this idea since it’s proposal. Monomeric, foreign proteins, from which aggregated protein had been carefully removed, are potent in their ability to inactivate B cells (56). This is paradoxical in terms of the sIg-cross linking model. Even more important to my mind was the idea that there should be minimal “physical” restraints on what self-antigens can inactivate their B cells. The crosslinking model implies that many self-antigens, monomeric in nature, would not inactivate their corresponding B cells, with substantial consequences for the generation of autoimmunity (56). I was greatly relieved to discover a few years ago that Michael Reth and others had shown that sIg self-aggregates in the membrane of B cells in the absence of antigen, and that small, monomeric antigens could disrupt such aggregation, initiating the generation of signal 1 (57). This discovery resolved the major uneasiness I had harboured for years concerning the two-signal model (56). Reth’s studies also accounted for the original observations on which the crosslinking model was based (57).

High-throughput screens can provide valuable information. It is bit like using genetic procedures to isolate an individual with a very rare phenotype. Both require much ingenuity and an underlying vision of why the screening will lead to significant findings. I recall the very extensive considerations that were necessary for Benzer and colleagues to isolate mutants of drosophila that impaired memory (58). This did not require these researchers to have a particular model as to how learning is achieved at the cellular/molecular levels, but rather the general idea that learning would be genetically controlled. This proposition was apparently not widely accepted at the time (58). I do understand that high-throughput procedures can pay considerable dividends. Much ingenuity can result in generating physiologically interesting observations. They can lead to inferences that conflict with other beliefs, and so to paradoxes. However, the mindless accumulation of data should not be misunderstood as progress.

These comments are in response to the reviewers’ philosophical reflections. I now address how various observations they brought up might impact the frameworks I favour. Some of the reviewers’ comments expressed incredulity that such observations were not addressed in my original submission. There were serious space limitations in my first submission. I wished to emphasize why I thought paradoxes are so important, and so minimized the description and discussion of ideas I favour. My apparent neglect of certain topics is thus understandable. I address why I think the comments are not warranted in view of my published papers. I respond in point form for ease of reference.

1. **The role of cytokines in determining the Th subset predominantly generated when antigen activates naive CD4 T cells.**

a) The class of immunity made in response to an antigen challenge is most often “coherent”. This coherence is reflected in different ways; for example, the IgG antibody produced at any one time usually predominantly belongs to one IgG subclass. The IgG subclass produced can change with time, and this represents a coherent switch in that the IgG subclass of most of the IgG antibodies change coordinately. It is clear how the coherence of the antibody response is realized (6). Consider two antibody epitopes of an antigen E, e_n_ and e_m_, for which there are separate B cells. Both B cells take in E and present the same diverse peptides of E to CD4 T cells. Thus, both B cells will receive the same spectrum of cytokines from E-specific Th cells. This spectrum determines the subclass of the IgG antibody produced (6, 59).

b) Coherence is also seen in the responses of CD4 T cells. The dose of simple and purified antigens determines the Th1/DTH or Th2/IgG nature of the ensuing response, as indicated above. A similar dependence is seen in the response to chemically complex antigens, such as mycobacteria and the intracellular, protozoan parasite, *L major*. Thus the Th1/Th2 phenotype of the response to the different chemical components of these complex antigens appears to be coordinately controlled (59).

It also seems physiologically important that the Th1/Th2 phenotype of simultaneous responses, to different antigens that do not cross-react, are normally independently determined, even when generated in the same lymphoid organ. If such independence did not obtain, then the Th1/Th2 phenotype of on-going immune responses, regularly occurring in mature individuals, would determine the Th1/Th2 phenotype of a primary response (6). We successfully tested this Principle of Independence. Briefly, we defined conditions where the iv injection of antigen A led to an exclusive Th1, splenic response, and conditions where similar injection of a non-crossreacting antigen, B, induced a predominant Th2, splenic response. We then injected both antigens from the same syringe iv into a mouse and examined the Th1/Th2 phenotype of the splenic responses to A and B. They were indistinguishable from those seen in singly immunized mice. This experiment, and its variants, support The Principle of Independence (60).

The Th1/Th2 phenotypes of responses to the different components of complex antigens appear to be coherently regulated, whereas the Th1/Th2 phenotypes of simultaneous responses to non-cross-reacting antigens, generated in the same lymphoid organ, appear to be normally independently determined. How can this be realized?

The Threshold Hypothesis readily accounts for independence. The strength of the antigen-dependent cooperation between CD4 T cells, mediated by antigen-specific B cells, is proposed to determine the Th1/Th2 phenotype (25). The Th1/Th2 phenotype of responses against non-cross-reacting antigens are independently determined as the CD4 T cell cooperation is mediated by distinct B cells (25). How is the Th1/Th2 phenotype of responses to different components of a chemically complex antigen, C, related? Consider two components, one much more prevalent, p, than the other, o. These components by themselves would obviously in general induce responses of different Th1/Th2 phenotype if given in amounts proportional to their presence in the chemically complex antigen. However, a B cell specific for p will present p peptides as well as some peptides derived from other components of C to which p is sometimes linked, as the linked components will be specifically taken up by the p-specific B cell, processed and presented. Similarly, a B cell specific for o will present peptides derived from o as well as peptides derived other components of C to which o is sometimes linked. In this way, Th cells specific for various linked components of C can influence the Th1/Th2 phenotype of the further generation of CD4 T cells specific for a given component of C. What might be the nature of this influence?

Cytokines produced by one Th subset, such as Th1 cells, often produce cytokines that support the further generation of Th cells belonging to this subset, and/or inhibit the further generation of CD4 T cells belonging to other subsets (59). Examples are the IFN-γ made by Th1 cells that inhibits the proliferation of Th2 but not of Th1 cells (61), and the IL-4 produced by Th2 cells that causes Th2 but not Th1 cells to divide (51). I have argued elsewhere that these and similar activities of different cytokines produced by Th cells result in a Th response, that consists predominantly of Th cells belonging to one Th subset, to evolve to become more dominated by this Th subset and so to become more coherent with time (6, 59). Thus, I suggest the Th1/Th2 phenotype of a response to a chemically complex antigen is initially approximately determined by the threshold mechanism, and this “decision” is sharpened up by the nature of the activities of the cytokines that Th cells of different Th subsets produce. I refer to this proposed sharpening up process as The Cytokine Implementation Hypothesis (59). It explains how responses can with time become more coherent, as delineated by Anne Kelsoe (62).

Lastly, I have discussed above how The Cytokine Milieu Hypothesis has led to investigations of the non-T cell source of the IL-4 believed to be required to initiate Th2 responses. We examined the in-vitro generation of IL-4 producing Th2 cells. This generation was inhibited by neutralizing anti-IL-4 antibody, as others had shown, resulting in a Th1 response. We showed that the IL-4 is produced by the CD4 T themselves (39). This confirms a prediction of the Cytokine Implementation Hypothesis, and confirms a conclusion reached by others on different grounds (63). One role I envisage for cytokines is to achieve coherence.

**2. The role of AIRE**

Our 1970 paper proposed that antigen could inactivate single naive lymphocytes, whereas their activation required antigen-mediated lymphocyte cooperation. We proposed that the time it took antigen to irreversibly inactivate a lymphocyte must not be shorter than the time it took lymphocytes to find each other and so initiate activation. If too short, no responses would ever be generated. If too long, then even very few lymphocytes may find and interact with one another before the lymphocytes were inactivated by the antigen, giving rise to autoimmunity (10).

It later became clear that central tolerance led to the elimination of most anti-self lymphocytes (7), and that our 1970 two signal model provided a possible explanation for peripheral tolerance. Moreover, not all “experimental antigens” could induce peripheral tolerance. For example, Colin Anderson and colleagues examined the basis of peripheral tolerance (64). They showed that a graft with one minor foreign histocompatibility antigen is not immunogenic but induces tolerance. In contrast, grafts with multiple minor differences were rejected. This is presumably because these graft-specific lymphocytes, the graft being more foreign than the graft with one minor difference, are produced and emigrate at a faster rate from the thymus and so cannot be irreversibly inactivated before they initiate the collaboration required for activation. These experiments supported our 1970 proposal, but also showed that if a “peripheral antigen is too foreign”, the rate of emigration of antigen specific T cells from the thymus is too rapid to allow peripheral tolerance to be established (65). Similarly, mice transgenic for a TcR that recognizes a peripheral self-antigen can be autoimmune, a result anticipated as lymphocytes specific for the antigen emigrate at such a fast rate from the thymus (66).

I now return to the considerations we discussed in our 1970 paper. If the time required to achieve irreversible inactivation is short, peripheral tolerance is favoured, but some lymphocytes specific for a foreign invader will be inactivated, to the detriment of the size and speed of the immune response to foreign invaders. If this time is relatively long, then there is time for antigen to mediate cooperation between lymphocytes when only few are present before lymphocyte inactivation occurs. This situation favours immunity and in extreme form results in autoimmunity, as seen in the Anderson experiments and in AIRE-deficient mice. I have argued that AIRE ensures that the rate of emigration of lymphocytes specific for AIRE-expressed “peripheral antigens” is lower than it would be in the absence of AIRE. With this lower rate, the time required to irreversibly inactivate peripheral lymphocytes can be longer and tolerance can still be maintained than the comparable length of time required in the absence of AIRE. This longer time means fewer lymphocytes specific for an invader will be inactivated and so responses to invaders can be faster and more intense (67). Note that AIRE expression does not eliminate all lymphocytes specific for peripheral antigens (8), such as insulin, but reduces their frequency, allowing them to be more reliably inactivated by the peripheral mechanism.

**3. The role of APC**

We suggested in 1970 that the interaction between specific lymphocytes might be facilitated by a third-party cell. This was admittedly not a classical APC as we know it today. However, by far the biggest challenge to our two signal concept arose when the MHC-restricted nature of the specificity of T cells was established and the role of APC became apparent. The challenge we faced, as proponents of two signal ideas, was our insistence on the importance that the interaction between lymphocytes, required for lymphocyte activation, involved the operational recognition of linked epitopes. Only in this case can the inactivation of lymphocytes by a peripheral self-antigen, pS, not be interfered with by a response to a non-crossreacting foreign antigen, F. As I have argued elsewhere, in the case of cooperation between CD4 T cells, operational recognition of linked epitopes can be achieved if an antigen-specific B cell mediates this cooperation (12, 13, 14, 65). The second paradox discussed above shows that the activation of MCC-specific CD4 T cells requires CD4 T cell cooperation mediated by an antigen-specific B cell (32). By the way, this challenging paper was published by Janeway’s laboratory!

4. One reviewer pointed out that the antigen Parish used in his studies, bacterial flagellin, may be extremely immunogenic because it expresses a PAMP that binds TLR5 (68). The reviewer suggested this might explain this antigen’s extreme immunogenicity. I agree. The important question for me, though, is whether this fact might make the phenomenon of low-zone, cell-mediated immune deviation an idiosyncratic phenomenon particular to this antigen. I suggest this is unlikely

Parish’s studies followed in design Mitchison’s, carried out with the foreign, vertebrate and therefore PAMP-free antigen, BSA, in mice (69). In both Mitchison’s and Parish’s experiments, repetitive pre-exposure to low and high amounts of antigen over several weeks inhibited the antibody response on the challenge, whereas medium doses led to priming for antibody. Parish showed in addition that the unresponsiveness for antibody production was associated with a state of DTH against the antigen. I suggest, as Parish also inferred (28), that Mitchison’s and his own observations reflect parallel phenomena. As BSA is PAMP-free, the expression of a PAMP is not necessary to establish low-zone cell-mediated immune deviation. We have also established “low zone cell-mediated immune deviation” to mycobacteria, to the intracellular, protozoan parasite, *Leishmania major*, and to transplantable tumors, as indicated above. These either express no or very different PAMPS, so the nature of the PAMPs in these cases appears not to be important in establishing low-zone cell-mediated immune deviation.

5. Most invaders, such as cancers and pathogens, multiply. One of the reviewers suggested I had neglected this point when I considered the nature of immune responses to such invaders and based my considerations on the dependence of the class of immunity on the dose of non-replicating antigens. I have attempted to address this issue in my publications. I summarise my point of view. Mitchison’s and Parish’s experiments, discussed above, led to the idea that chronic exposure over weeks, to relatively low and high doses of non-replicating antigens, could result in a state of cell-mediated immune deviation. Challenge of such exposed animals to a challenge that produces predominant IgG antibody in unexposed animals resulted in little antibody production but in sustained DTH.

We tried to imagine the pertinence of such observations, employing non-multiplying antigens, to real life situations. Infection of an animal with one very rapidly multiplying organism can result in billions of organisms in a week. Such an infection, not surprisingly, results in rapid IgG antibody production (6). Consider a slowly growing cancer cell, microorganism, or parasite. In this case, a cell-mediated response may be initially induced and, particularly if the slowly multiplying invader is susceptible to cell-mediated attack, one might well imagine the response to be able to both contain the invader, at a stable level, and be locked into a cell-mediated mode, a la Parish (6). This is the basis of our low dose vaccination strategy described above, and tested against tumors, and intracellular mycobacteria and protozoa.

6. One reviewer pointed out that the BCG vaccine provides similar protection over a broad range of doses, from ten to a million organisms (70). The reviewer wonders how I might explain the protection flowing infection with high numbers of BCG. It should be noted that this finding was made in an inbred strain of guinea pigs with a particular strain of BCG.

There are two issues pertinent to this question that we had to face in imagining how to realize our low dose vaccination strategy against invaders preferentially susceptible to cell-mediated immunity. 1. Does the “dose rule” hold in genetically diverse individuals? 2. How to make a vaccine that is universally efficacious in genetically diverse individuals? We explored the first question in the murine model of infection by Leishmania major. Many had studied infections in different strains of mice. We infected different strains of mice with widely different numbers of slowly multiplying parasites, all by the same route. We found that infection with relatively low numbers gave rise to sustained Th1 responses, and with higher numbers to responses that with time evolved into a predominant Th2 mode. This general finding supports a positive answer to the first question: the dosage rule generally holds. We were able to define a transition number for each mouse strain, n_t_. Infection with a number of parasites below n_t_ resulted in a stable Th1 response and, above n_t_, resulted in a response that developed in time a substantial Th2 component. Infection with numbers considerably larger than n_t_ resulted in a rapid Th2 response. We found the value of n_t_ to vary over a 100,000 fold range in different strains of mice (34).

We suggest that infection of a population of animals or people, with a slowly growing organism, such as BCG, with a number below the n_t_ for all individuals, will in time generate a Th1 response and a Th1 imprint in all the individuals, and so would likely constitute universally efficacious vaccination (6, 34).

To return to the observation that infection with ten to a million BCG all induced protective immunity in a particular strain of guinea pigs. The accepted inference is infection with both low and high numbers of organisms induce Th1 immunity. In the case of *L major*, we found n_t_ to be 5x10^8^ in CBA mice. Those mice given this number, that developed disease, produced antibody and generated Th2 cells (34). Thus, infection of CBA mice with both low and high numbers (<5x10^8^) of parasites reliably induces Th1 responses. I think the concept of a transition number helps to resolve this paradox. It is possible, in individuals with exceptionally high n_t_, to find they generate a Th1 response against a slowly growing microorganism when infected with very different numbers of organisms Also, the finding that n_t_ can vary so profoundly in different individuals may help explain why it has been so difficult to find a universally efficacious vaccination strategy against tuberculosis (47). It also provides, as indicated above, the context to formulate such a strategy.

7. Another question raised was how could the importance of antigen dose, in determining the class of immunity generated, be modified by the coadministration, close to the time of immunization, of drugs? The particular observation cited was the ability of cyclophosphamide to modulate a response to SRBC from a humoral to a cell-mediated mode (71, 72). We suggest this occurs because the drug kills most dividing cells, including CD4 T cells, and therefore acts to modulate the response, in accord with the threshold mechanism (6, 73, 74).

I would like to finish with a story about the second book on immunology that I wrote, entitled “The Foundations of Immunology and Their Pertinence to Medicine” (6). I attempted to make this book short and accessible to the layperson. It is about 180 pages long. I am aware that some people find my writing heavy going. Given this, I wanted readers to feel they were getting somewhere as they tried to turn the pages. I asked my editor to use as large a size of print as possible. She agreed. She explained that any further increase in print size meant libraries would only offer the book in the kid’s section. Hopefully, less is sometimes more!

## Data availability statement

The original contributions presented in the study are included in the article/Supplementary Material. Further inquiries can be directed to the corresponding author.

## Author contributions

The author confirms being the sole contributor of this work and has approved it for publication.

## Acknowledgments

I thank Calliopi Havele and Mirek Cygler for comments on an earlier draft and Callioipi Havele for further comments on this draft.

## Conflict of interest

The author declares that the research was conducted in the absence of any commercial or financial relationships that could be construed as a potential conflict of interest.

## Publisher’s note

All claims expressed in this article are solely those of the authors and do not necessarily represent those of their affiliated organizations, or those of the publisher, the editors and the reviewers. Any product that may be evaluated in this article, or claim that may be made by its manufacturer, is not guaranteed or endorsed by the publisher.

## Abbreviations

CFA, compete Freund’s adjuvant; CTL, cytotoxic T lymphocyte; DAMP, danger-associated molecular pattern; MCC, mouse cytochrome C; PAMP, pathogen-associated molecular pattern.
